# ERK1/2 drives IL-1β-induced expression of TGF-β1 and BMP-2 in torn tendons

**DOI:** 10.1038/s41598-019-55387-1

**Published:** 2019-12-12

**Authors:** Wataru Morita, Sarah J. B. Snelling, Kim Wheway, Bridget Watkins, Louise Appleton, Andrew J. Carr, Stephanie G. Dakin

**Affiliations:** 1 Botnar Research Centre, Nuffield Department of Orthopaedics, Rheumatology and Musculoskeletal Sciences (NDORMS), University of Oxford, Windmill Road, Oxford, UK; 2 NIHR Oxford Biomedical Research Centre, Botnar Research Centre, NDORMS, University of Oxford, Windmill Road, Oxford, OX3 7LD UK

**Keywords:** Mechanisms of disease, Translational research, Musculoskeletal abnormalities, Mechanisms of disease, Musculoskeletal abnormalities

## Abstract

Diseased and injured tendons develop fibrosis, driven by factors including TGF-β, BMPs and CTGF. IL-1β and its signal transducer Erk1/2 are known to regulate TGF-β expression in animal tendons. We utilised tissues and cells isolated from patients with shoulder tendon tears and tendons of healthy volunteers to advance understanding of how inflammation induces fibrosis in diseased human tendons. ERK1/2 expression was reduced in torn (diseased) compared to healthy patient tendon tissues. We next investigated the fibrotic responses of tendon-derived cells isolated from healthy and diseased human tendon tissues in an inflammatory milieu. IL-1β treatment induced profound ERK1/2 signalling, *TGFB1* and *BMP2* mRNA expression in diseased compared to healthy tendon-derived cells. In the diseased cells, the ERK1/2 inhibitor (PD98059) completely blocked the IL-1β-induced *TGFB1* and partially reduced *BMP2* mRNA expression. Conversely, the same treatment of healthy cells did not modulate IL-1β-induced *TGFB1* or *BMP2* mRNA expression. ERK1/2 inhibition did not attenuate IL-1β-induced *CTGF* mRNA expression in healthy or diseased tendon cells. These findings highlight differences between ERK1/2 signalling pathway activation and expression of TGF-β1 and BMP-2 between healthy and diseased tendon tissues and cells, advancing understanding of inflammation induced fibrosis during the development of human tendon disease and subsequent repair.

## Introduction

Musculoskeletal diseases contribute a significant global burden^1,2^ by causing pain and disability impacting upon activities of daily living and quality of life^3,4^. Tendon diseases account for one third of all musculoskeletal complaints to a general practitioner^5^ with the supraspinatus tendon of the rotator cuff being frequently affected^6,7^. Diseased tendons are characterised by fibrosis, the inappropriate and often excessive production of extracellular matrix (ECM) proteins^8^. Fibrotic tendons are biologically and mechanically inferior compared to healthy tendons, predisposing the diseased tendon to tear^9,10^. Transforming growth factor beta (TGF-β) and its closely related growth factors including connective tissue growth factor (CTGF) and bone morphogenetic proteins (BMPs) have important roles in cell proliferation, differentiation and ECM metabolism^11,12^. These factors are implicated as mediators of fibrosis by studies of fibrotic diseases in other organs. TGF-β is regarded as the key fibrotic mediator, and its increased expression has been shown in animal studies of tendon overuse and injury healing^13^. CTGF has gained interest as a downstream regulator of TGF-β^13^. Dysregulated expression of BMPs such as BMP-2, BMP-7 and BMP-12 has also been reported in relation to tendon injury and healing^13^, implicating their involvement in tendon fibrosis.

Fibrosis develops as a result of dysregulated and persistent inflammation^14^, and an increasing number of studies support the regulatory roles of inflammatory cytokines in the development and progression of a tendon disease^3,15,16^. The effects of inflammatory cytokines on the expression of ECM-related genes and proteins have been investigated in both human and animal tendon-derived cells^17^. Tendon-derived cells of injured rat patellar tendons show an altered response to IL-1β treatment compared to those of un-operated tendons *in vitro*^18^. IL-1β is a pro-inflammatory cytokine known to take part in early stage human rotator cuff tendon disease^19^. Previous works from our group have also identified that tendon-derived cells from diseased human rotator cuff tendons show increased responsiveness to pro-inflammatory cytokines^19,20^ and increased expression of IL-1 receptor in diseased human rotator cuff tendon tissues^21^. IL-1β activates mitogen-activated protein kinases (MAPKs) such as the extracellular signal-regulated kinase (ERK) 1/2^22^. Activation of the Erk1/2 signalling pathway has been implicated in a rat supraspinatus tendinopathy model^23^ and in regulating *Tgfb1* mRNA expression in rat Achilles tendon-derived cells^24^. However, to date no studies have investigated how inflammatory cytokines regulate fibrotic mediators including TGF-β, CTGF and BMP during the development of a human tendon disease and subsequent repair.

The aim of this study was to identify the mechanism by which inflammatory cytokines regulate fibrotic mediators including TGF-β, CTGF and BMP in tendon-derived cells of healthy and diseased patient tissues. We focused on large to massive tears for the diseased cohort as they represent end-stage disease and established pathology where the tendon has undergone fibrotic changes. We hypothesised that the ERK1/2 signalling pathway regulates IL-1β-induced expression of these fibrotic mediators in tendon-derived cells isolated from patients with end-stage torn rotator cuff tendons.

## Results

### Diseased tendon tissues show reduced levels of active ERK1/2

**Figure 1:**
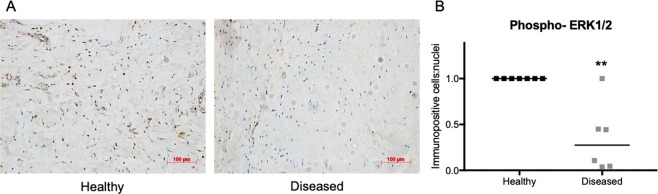
Expression of phosphorylated (phospho-) ERK1/2 is reduced in torn (diseased) supraspinatus tendon tissues. (**A**) Representative images of healthy and diseased (large to massive tear) supraspinatus tendon longitudinal sections with haematoxylin (blue) for nuclei and DAB (brown) for immunopositive staining. Scale bar = 100 μm. (**B**) Semi-quantitative analysis of levels of protein expression quantified as the number of immunopositive cells relative to the number of nuclei. Protein levels of phosphorylated ERK1/2 was reduced in diseased (N = 6) compared to healthy (N = 7) tendon tissues. Bars shown represent median. *Indicates significant difference to the healthy group. *P < 0.05, **P < 0.01.

We investigated the differences in the expression of phosphorylated ERK1/2 between supraspinatus tendon tissues collected from healthy volunteers and patients with diseased tendons (large to massive tears) by immunohistochemistry (Fig. 1). Semi-quantitative analysis of immunopositive staining showed a 3.6-fold decreased expression of phosphorylated ERK1/2 in diseased compared to healthy tendons (P = 0.0047).

### Diseased tendon-derived cells show increased ERK1/2 signalling pathway activation by IL-1β treatment

**Figure 2:**
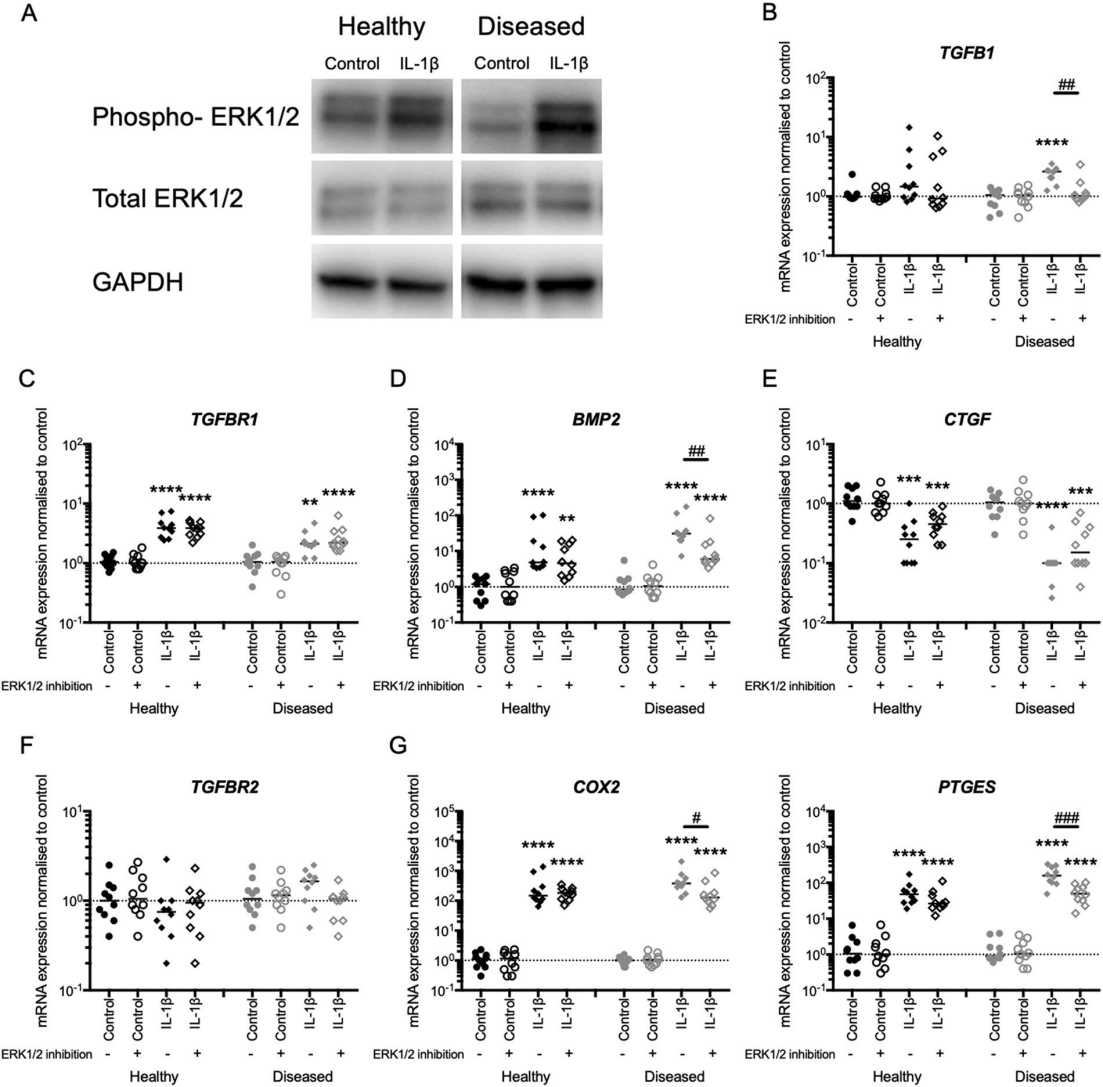
ERK1/2 pathway drives IL-1β-induced *TGFB1* and *BMP2* mRNA expression in tendon-derived cells of torn tendons (diseased cells). (**A**) Western blotting for phosphorylated (phospho-), total ERK1/2 and GAPDH indicated increased induction of ERK1/2 signalling pathway activation in response to IL-1β but not vehicle control (control) treatment in diseased (N = 3) compared to healthy (N = 3) cells. Representative blots are shown. (**B**–**G**) Healthy (N = 10) and diseased (N = 10) tendon-derived cells were treated with IL-1β or vehicle control (control) with or without ERK1/2 inhibition. mRNA expression was quantified by RT-qPCR. (**B**) ERK1/2 inhibition completely suppressed IL-1β-induced *TGFB1* mRNA expression in diseased cells. (**C**) ERK1/2 inhibition did not modulate IL-1β-induced *TGFBR1* mRNA expression in either cell group. (**D**) ERK1/2 inhibition partially suppressed IL-1β-induced mRNA expression of *BMP2* in diseased cells only. (**E**) ERK1/2 inhibition did not modulate IL-1β-induced *CTGF* mRNA expression in either cell group. (**F**) IL-1β treatment did not induce *TGFBR2* mRNA expression in either cell group. (**G**) ERK1/2 inhibition partially suppressed IL-1β-induced mRNA expression of NF-κB signalling pathway target genes *PTGES* and *COX2* in diseased cells only. Bars shown represent median. *Indicates significant difference to the respective vehicle control. ^#^Indicates significant difference between with and without ERK1/2 inhibition. ^*/#^P < 0.05, ^**/##^P < 0.01, ***P < 0.001, ****P < 0.0001.

Having shown diminished tissue expression of active ERK1/2 in diseased tendon tissues, we next investigated whether IL-1β treatment induces ERK1/2 signalling pathway activation in tendon-derived cells of healthy (healthy cells) and torn (large to massive tear) supraspinatus tendons (diseased cells) in an *in vitro* cell culture model. IL-1β treatment (5 ng/ml) for 30 minutes induced expression of phosphorylated ERK1/2 in both healthy and diseased cells determined by Western blotting. Representative images of the blots for healthy and diseased cells are shown in Fig. 2A. Semi-quantitative analysis of the blots indicated increased ERK1/2 signalling pathway activation in the diseased compared to healthy cells (Supplementary Fig. 1).

### ERK1/2 drives *TGFB1* and *BMP2* expression in IL-1β stimulated diseased tendon-derived cells

Having shown activation of ERK1/2 signalling by IL-1β in tendon-derived cells, we sought to establish firstly whether IL-1β induces expression of the fibrotic mediators including TGF-β1, CTGF and BMPs (BMP-2, BMP-7 and BMP-12), and to determine whether induction of these fibrotic mediators was mediated via ERK1/2 signalling. IL-1β treatment (5 ng/ml) for 24 hours induced *TGFB1* mRNA expression compared to vehicle control in diseased cells only (P < 0.0001) (Fig. 2B). This treatment also induced *TGFBR1* and *BMP2* mRNA expression compared to vehicle control in both healthy (P < 0.0001 and P < 0.0001, respectively) and diseased cells (P = 0.0011 and P < 0.0001, respectively) (Fig. 2C,D). IL-1β treatment suppressed *CTGF* mRNA expression compared to vehicle control in healthy and diseased cells (P = 0.0002 and P < 0.0001, respectively) (Fig. 2E), but did not modulate *TGFBR2* mRNA expression in both cells (Fig. 2F). mRNA expression of *BMP7* and *BMP12* were low-level regardless of treatment in both healthy and diseased cells. Having identified the regulatory role of IL-1β in the mRNA expression of *TGFB1*, *TGFBR1*, *BMP2* and *CTGF*, we next investigated the consequences of ERK1/2 pathway inhibition on the IL-1β-induced expression of these fibrotic mediators using the ERK1/2 inhibitor PD98059 (8 μg/ml, pre-treatment for 1 hour)^25^. ERK1/2 pathway inhibition diminished IL-1β-induced *TGFB1* mRNA expression in diseased cells (P = 0.0021) (Fig. 2B), but did not attenuate *TGFBR1* or *CTGF* mRNA expression in healthy or diseased cells (Fig. 2C,E). ERK1/2 pathway inhibition partially suppressed the IL-1β-induced *BMP2* mRNA expression in diseased but not healthy cells (P = 0.0019) (Fig. 2D). BMP-2 is a target gene of the NF-κB pathway^26–29^ and similar effects of ERK1/2 pathway inhibition were observed in the IL-1β-induced mRNA expression of additional NF-κB target genes *PTGES* and *COX* in diseased cells (P = 0.0005 and P = 0.0232, respectively) but not healthy cells (Fig. 2G). Taken together these results demonstrate that the ERK1/2 signalling pathway drives the abnormal expression of TGF-β1 and BMP-2 in response to IL-1β treatment in diseased tendon-derived cells.

### ERK1/2 pathway inhibition suppresses IL-1β-induced BMP-2 but not TGF-β1 cell signalling activity

**Figure 3:**
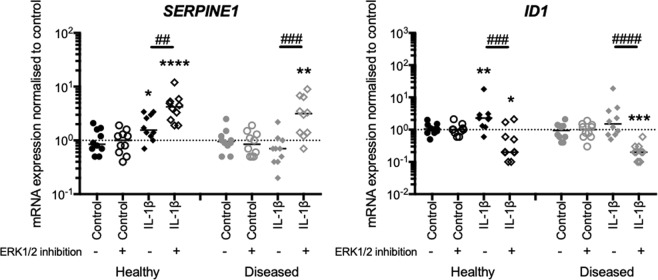
ERK1/2 pathway inhibition suppresses IL-1β-induced BMP-2 but not TGF-β1 cells signalling activity. Healthy (N = 10) and diseased (N = 10) tendon-derived cells were treated with IL-1β or vehicle control (control) with or without ERK1/2 inhibition. mRNA expression was quantified by RT-qPCR. IL-1β-induced canonical TGF-β and BMP signalling pathway activities were diminished in diseased cells. ERK1/2 inhibition up-regulated IL-1β-induced *SERPINE1* mRNA expression in both cells. ERK1/2 inhibition down-regulated IL-1β-induced *ID1* mRNA expression in both cells. Bars shown represent median. *Indicates significant difference to the respective vehicle control. ^#^Indicates significant difference between with and without ERK1/2 inhibition. ^*/#^P < 0.05, ^**/##^P < 0.01, ^***/###^P < 0.001, ^****/####^P < 0.0001.

Having shown that ERK1/2 signalling drives IL-1β-induced TGF-β1 and BMP-2 expression in diseased tendon-derived cells, we next investigated the involvement of the ERK1/2 signalling pathway in the IL-1β-induced TGF-β1 and BMP-2 cell signalling activities by measuring their canonical SMAD signalling pathway target gene *SERPINE1*^30^ and *ID1*^31^ expression, respectively. Serpin E1 (serpin family E member 1) also known as plasminogen activator inhibitor 1 represses the plasmin-dependent protease activities and negatively regulates the breakdown of ECM collagens and fibrins resulting in fibrosis^32^. ID-1 (inhibitor of DNA-binding 1) is a pleiotropic transcription factor taking part in cell growth, differentiation and angiogenic activities^33^, and also counteracts TGF-β1, subsequent collagen production and hence fibrosis^34^. IL-1β treatment induced *SERPINE1* and *ID1* mRNA expression compared to vehicle control in healthy cells only (P = 0.0330 and 0.0063, respectively). However, when ERK1/2 signalling was inhibited, IL-1β elicited a significant induction in *SERPINE1* mRNA expression compared to IL-1β treatment alone in both healthy and diseased cells (P = 0.0048 and 0.0005, respectively). In contrast, ERK1/2 pathway inhibition down-regulated IL-1β-induced *ID1* mRNA expression in both healthy and diseased cells (P = 0.0004 and P < 0.0001, respectively) (Fig. 3). These results identify that IL-1β stimulated diseased cells show diminished TGF-β1 and BMP-2 SMAD cell signalling activities. The ERK1/2 pathway may have differential roles in IL-1β-induced TGF-β1 and BMP-2 SMAD cell signalling activities.

## Discussion

Dysregulated repair and inflammation drive fibrosis in a diseased tendon^3,14,15^. Numerous animal studies of tendon overuse and injury healing have focused on TGF-β and its closely related CTGF and BMPs as mediators of fibrosis^13^. This study shows for the first time that diseased human tendon-derived cells exhibit dysregulated gene expression of fibrotic mediators in response to IL-1β treatment compared to healthy tendon-derived cells.

We investigated if ERK1/2 signalling pathway contributes to the IL-1β-induced *TGFB1* and *BMP2* expression in diseased cells. IL-1β treatment induced *TGFB1* in diseased cells. Increased Erk1/2 signalling pathway activation in relation to tendon overuse and Erk1/2-driven *Tgfb1* mRNA expression have been reported in studies using rat tendons^23,24^. In the current study, ERK1/2 signalling pathway inhibition negated the IL-1β-induced *TGFB1* mRNA expression, and also partially suppressed the IL-1β-induced *BMP2* mRNA expression in diseased cells. These results indicate the involvement of the ERK1/2 signalling pathway in end-stage tendon disease. ERK1/2 signalling pathway has been reported to suppress the expression of NF-κB pathway target genes in a monocytic cell line (THP-1)^35^. The cellular effects of IL-1β treatment *in vitro* may differ between cells isolated from clinical samples and cell lines. Furthermore, immunostaining in this study showed that the level of active ERK1/2 is significantly reduced in diseased compared to healthy tendon tissues. Reduced expression of fibrotic proteins in tissue samples from patients with chronic supraspinatus tendon tears has also been reported^36^. Increased responsiveness to inflammatory stimuli in diseased compared to healthy human tendon-derived cells^19,20^ and increased tissue expression of their receptors in diseased tendons have been shown^21^. Whilst diseased tendon tissues showed reduced expression of TGF- β1 and BMP-2 proteins, increased IL-1β-induced expression of *TGFB1* and *BMP2* by diseased cells may be attributable to this capacity for increased responsiveness to inflammatory stimuli. Increased responsiveness to inflammatory stimuli may subsequently influence fibrotic healing during tendon repair. The nature of the IL-1β-induced expression of fibrotic mediators driven by the ERK1/2 signalling pathway in torn human tendons is likely to differ from the pathobiology of increased Erk1/2 signalling pathway activation noted in a rat model of supraspinatus tendinopathy^23^.

IL-1β signal transducers such as MAPKs and NF-κB suppress TGF-β- and BMP-induced SMAD activities^37–43^. Hence, the increased ERK1/2 and NF-κB signalling pathway activation (indicated by the up-regulated expression of NF-κB target genes) could be contributing to the diminished IL-1β-induced TGF-β1 and BMP-2 cell signalling activities in diseased cells. TGF-induced factor (TGIF) is an active transcriptional co-repressor of the canonical TGF-β signalling pathway, induced by ERK1/2 signalling pathway activation in fibroblasts^44^. IL-1β treatment induced *TGIF1* mRNA expression in healthy and diseased cells, which was partially suppressed by ERK1/2 pathway inhibition in diseased cells only (Supplementary Fig. 2). ERK1/2 regulation of TGIF expression at the transcriptional level has not been reported thus far^44,45^. ERK1/2 signalling pathway inhibition affected IL-1β-induced mRNA expression of TGF-β1 and BMP-2 target genes *SERPINE1* and *ID1*, respectively in both healthy and diseased cells. The results of our study highlight the complexity of the interactions between MAPKs, NF-κB and SMAD signalling pathways, which also could be cell-type specific^46–48^.

CTGF is a downstream regulator of TGF-β^49^, which has been implicated in other fibrotic diseases^50–52^. CTGF and IL-1β are both expressed in tendon overuse animal models^53^, but our results showed that IL-1β treatment suppresses *CTGF* mRNA expression in human tendon-derived cells *in vitro*. Similar cell culture experiments treating rat mesangial cells, human dermal fibroblasts, human healthy and diseased (dihydropyridine-induced gingival overgrowth) gingival fibroblasts with IL-1β all show inconsistent results in *CTGF* mRNA expression between studies^54–56^. The effects of IL-1β on *CTGF* mRNA expression is likely cell and context specific, and the interpretation of the results from this study should not be expanded beyond the comparison of human tendon-derived cells of healthy and torn tendons.

We acknowledge that there are limitations in this study. The cells utilised for the experiments may include a variety of cells such as tendon fibroblasts (tenocytes), tendon stem or progenitor cells owing to the cell isolation and culture method^57^. Hence, we collectively termed these cells as ‘tendon-derived cells’^58,59^. The donors of diseased tendons were significantly older than those of healthy tendons, and other studies have indicated changes in the activity of ERK1/2 and NF-κB signalling pathways in relation to aging and senescence^60,61^. Increased responsiveness of diseased human tendon-derived cells to inflammatory stimuli^20^ have also been related to aging^62,63^. There is a possibility that our results are confounded by age-related changes. Future studies addressing whether the diminished tissue expression of active ERK1/2 and the increased IL-1β-induced activation is related to aging or represents end-stage tendon disease is warranted. IL-1β activates other signalling pathways such as p38 and SAPK/JNK, and how they interact will also require further investigation. Moreover, IL-1β and the subsequent expression of fibrotic mediators may act on cell morphology, cytoskeleton, proliferation, vascularity and the ECM in the development of fibrosis, which were not addressed by this study. Healthy cells for the *in vitro* experiments were derived from an anatomically different hamstring tendon. There are limitations in using functionally distinct tendons as a comparator, but it is a positional tendon alike the rotator cuff that has been used as an ethically feasible source to obtain ‘healthy’ tendon samples^36,64^, and this would be preferable to using cadaveric tissue where the health status of the donor tissue is not known^21^. Furthermore, our study sampled diseased tendons tissues from patients with large to massive supraspinatus tears. Histological and molecular characteristics of torn tendon tissues may differ according to the chronicity of the tear^65^, and hence the findings of this study should only be applicable to the understanding of end-stage disease of large to massive supraspinatus tendon tear.

**Figure 4:**
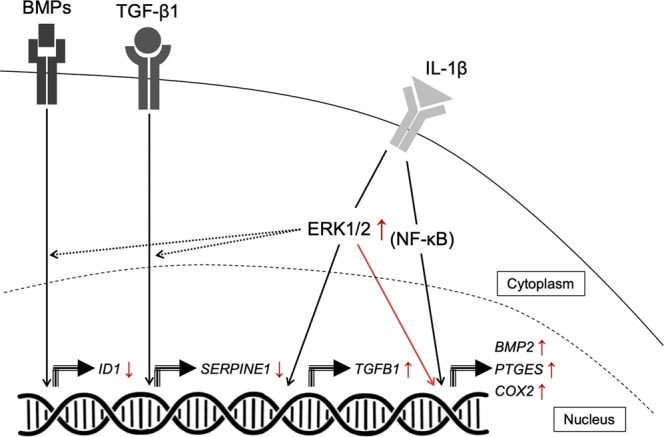
IL-1β induces differential expression of TGF-β1 and BMP-2 in diseased tendon-derived cells. Only the genes with significant differences between healthy and diseased cells are shown. Black arrows indicate the normal conditions healthy cells. Red arrows indicate the significant findings in diseased cells. IL-1β treatment induced *TGFB1* mRNA expression in diseased cells. IL-β treatment induced mRNA expression of *BMP2* and NF- κB target genes in healthy and diseased cells, which was up-regulated in the diseased cells in relation to ERK1/2 signalling. IL-1β-induced TGF-β target gene *SERPINE1* and BMP target gene *ID1* expression was diminished in diseased cells.

The findings of this study suggest that the profound ERK1/2 signalling pathway activation in response to IL-1β treatment regulates the increased *TGFB1* and *BMP2* mRNA expression in the diseased cells. Contrarily, canonical TGF-β1 and BMP-2 cell signalling activities measured by the mRNA expression of their target genes were diminished in IL-1β-induced diseased cells (Fig. 4). Tissue expression of active ERK1/2 is also reduced in diseased compared to healthy tendons. These findings highlight differences between ERK1/2 signalling pathway activation and expression of TGF-β1 and BMP-2 between healthy and diseased human tendon tissues and cells, advancing the understanding of fibrosis in diseased human tendons. Improved understanding of inflammation-induced fibrotic pathways should inform future strategies to therapeutically target tendon fibrosis.

## Materials and Methods

### Study approval

Healthy and diseased tendon tissues were obtained under ethical approval from the Oxford Musculoskeletal Biobank (Oxfordshire REC C 09/H0606/11) or by the local or multicentre research ethics committees (OUH R&D REC ref. 15/WS/0266, South Central Oxford REC B ref. 14/SC/022, and UK NHS RECs ref 10/H0402/24). This study was carried out in concordance with the United Kingdom Human Tissue Act and the Declaration of Helsinki. All patients gave informed consent prior to donating their tissue.

### Collection of human tendon tissues

All patients were recruited in shoulder referral clinics. The structural integrity of the supraspinatus tendon was confirmed by high-definition ultrasound examination^66^. The size of a torn supraspinatus tendon was measured intraoperatively in the longest diameter, and defined as small, medium, large or massive^67^. The exclusion criteria were prior history of diseases of the affected shoulder; other shoulder diseases that do not relate to the rotator cuff such as osteoarthritis, idiopathic frozen shoulder and fracture; bilateral shoulder problems; systemic inflammatory diseases; and symptoms related to the cervical spine or neurological disorders. Clinical diagnoses were not confirmed by pathology.

Diseased supraspinatus tendon tissues were obtained from the edge of the torn tendon as part of the debridement procedure of a repair surgery in patients operated for large to massive supraspinatus tears. Tissues from 7 male and 3 female patients aged 62.7 ± 7.8 years were used for explant culture. Tissues from 4 male and 2 female patients aged 64.8 ± 5.5 years were used for tissue protein expression analysis.

Healthy tendon tissues for explant culture were obtained from surplus hamstring tendons of 6 male and 4 female patients aged 24.6 ± 7.7 years going under autograft anterior cruciate ligament reconstruction surgery. Healthy supraspinatus tendon tissues for tissue protein expression analysis were obtained from 6 male and 1 female patients aged 34.7 ± 16.6 years by a validated biopsy technique using a long core biopsy instrument (Bard) under ultrasound guidance and local anaesthesia^68^. There were no adverse patient effects reported for this procedure.

### Immunohistochemistry for phosphorylated ERK1/2 in healthy and diseased tendon tissues

Fresh tendon tissue samples were immediately fixed in 10% neutral-buffered formalin and left for the fixative to infiltrate sufficiently. Fixed tissues were processed using ASP300 S tissue processor (Leica) and embedded in paraffin wax. Sections 5 μm thick were sliced using RM2125 RTS manual rotary microtome (Leica), collected onto glass slides, dried at 60 °C for 30 minutes and then 37 °C for 60 minutes. Tissue sections were baked at 60 °C for 60 minutes and then placed in an automated pre-treatment system (PT Link, Dako). Autostainer Link 48 (Dako) was used for antibody staining and EnVision FLEX DAB+ Chromogen System (Dako) for visualisation of its binding to the antigen using 3,3′- diaminobenzidne (DAB). Haematoxylin was used for counterstain. The procedures were performed according to the manufacturer’s instructions. A rabbit polyclonal anti-phospho-ERK1/2 antibody (Abcam) was used at a dilution of 1:1000 after validation using sections of human tendon tissues. After staining, slides were taken into graded industrial methylated spirit (twice, 3 minutes each) for dehydration, then xylene (twice, 5 minutes each) for clearance and mounted (DPX Mounting medium, Fisher Scientific).

Ten to 20 images of each stained slides were captured using LSM 700 inverted brightfield microscope (Carl Zeiss MicroImaging) and Axiovision Rel. 4.8 imaging software (Carl Zeiss) at 100x magnification with oil immersion. Attention was paid so that the images did not overlap. Histological fibrotic changes (e.g. cell morphology, cellularity, disorganised ECM and vascularity) were confirmed in the diseased tissues, but were not quantified as this was not the objective of this study. The number of DAB and haematoxylin stained cells in each image was counted using ImageJ using a previously validated algorithm, with a manually set threshold for each dataset^69–71^. Semi-quantitative analysis of immunopositive cells by immunohistochemistry was carried out by calculating the percentage of DAB positive cells relative to the total number of cell nuclei imaged by haematoxylin. The details of the macros used are shown in Supplementary Method 1.

### Isolation and culture of tendon-derived cells

Cells were isolated from tendon tissues and cultured based on protocols described previously^19,20^. In detail, fresh samples of tendon tissues were immediately incised into pieces 1 to 2 mm large^3^ and cultured in media containing 50% foetal bovine serum (FBS; Labtech), Dulbecco’s Modified Eagle Medium/Nutrient Mixture (DMEM F-12; Fisher Scientific) and 1% penicillin-streptomycin (PS; Lonza) at 37 °C and 5% CO_2_. After confirming by light microscopy that the cells had migrated out from the tissue after 7 to 10 days, culture media were changed to 10% FBS, DMEM F-12 and 1% PS until the cells were sub-confluent (approximately 80 to 90% confluent) for further passage or frozen storage. Cells were suspended in 90% FBS and 10% dimethyl sulfoxide (Sigma Aldrich) and stored at −80 °C for short-term (up to 3 months) and liquid nitrogen (approximately −200 °C) for long-term storage^72^. Our group has previously shown that these cells are negative for the leukocyte marker CD45 and vascular markers CD31 and CD34 by fluorescence-activated cell sorting and immunohistochemistry^20,73^. Culture media were changed twice a week. Cells cultured up to passage 2 or 3 were used for experiments^74^.

### Culture and treatment of tendon-derived cells with IL-1β

Healthy (N = 10) and diseased (N = 10) tendon-derived cells were seeded in 24-well plates at a density of 30,000 cell/ml (0.5 ml) and cultured to sub-confluence for the mRNA expression study. For analysis of cell signalling pathway activation by Western blotting, healthy (N = 3) and diseased (N = 3) cells were seeded in 6-well plates at a density of 60,000 cells/ml (2 ml) and cultured to sub-confluence. Following serum starving overnight, the cells were pre-treated with a small molecule inhibitor of the ERK1/2 pathway (PD98059; 8 μg/ml (30 μM)) for 1 hour prior to addition of human recombinant IL-1β (5 ng/ml; Merck Millipore) in serum-free medium or vehicle control (serum-free medium). The treatment dosage was determined by a validation experiment (Supplementary Method 3). Cells from both cohorts were treated for 24 hours and 30 minutes for the mRNA expression and cell signalling pathway activation analysis experiments, respectively. Vehicle only treatments served as experimental controls.

### mRNA expression analysis of cell lysates

mRNA was isolated from tendon-derived cells using RNeasy Minikit (Qiagen) with treatment by DNase I (Thermo Scientific). NanoDrop 1000 spectrophotometer (Thermo Scientific) was used to confirm the quality of the processed samples to be within 1.8 and 2.2 by the ratio of absorbance at 260 nm and 280 nm. mRNA was reverse transcribed into cDNA using High-Capacity cDNA Reverse Transcription Kit (Applied Biosystems) and a ribonuclease inhibitor (RNasin Plus RNase Inhibitor; Promega). The generated cDNA was further diluted with nuclease-free water resulting in 0.5 to 2.5 ng μl^−1^ ^19^.

Real-time quantitative polymerase chain reaction (RT-qPCR) was performed using a ViiA 7 Real-Time PCR System (Applied Biosystems) using SYBR Green Master Mix (Applied Biosystems) and validated primers from QuantiTect Primer Assays (Qiagen) or Primerdesign (Southampton, UK). The details of the primers used and their sequences are shown in Supplementary Table 1. Relative expression was calculated by the comparative CT method with a set threshold was of 0.15 for all genes^75^. All of the experiments and measurements were performed in technical duplicates, and the average CT values were used for analysis with a coefficient of variation between duplicates less than 0.05. β-actin and glyceraldehyde-3-phosphate dehydrogenase (GAPDH) were used as endogenous housekeepers, which the geometric mean of their CT values was used for analysis^76^.

### Cell signalling pathway activity analysis

Tendon-derived cells were treated with 1 mM sodium orthovanadate (Sigma-Aldrich) for phosphatase inhibition and then scraped in a cell lysis buffer that contains 0.5 mM sodium orthovanadate, 32 mM Tris base (Fisher Scientific), 50 mM (5%) glycerol (Sigma-Aldrich), 34.7 mM (1%) sodium dodecyl sulphate (SDS; Fisher Scientific) and 0.5% Protease Inhibitor Cocktail Set III (Merck Millipore). Protein separation was performed by electrophoresis by loading approximately 2.5 μg of protein per well to 10% polyacrylamide gel electrophoresis gels (BioRad). The amounts of the proteins loaded were measured by a bicinchoninic acid assay. Proteins were transferred from gels on to a 0.2 μm polyvinylidene difluoride membrane (BioRad) using a Western blot transfer system (BioRad). Molecular weight was indicated by Precision Plus Protein Dual Color Standards (BioRad). The details of the antibodies used and their working dilutions are shown in Supplementary Table 2. GAPDH was used as the endogenous housekeeper. Anti-rabbit IgG antibody was used as the secondary antibody. Proteins were detected by enhanced chemiluminescence (GE Healthcare Life Sciences) and visualised using Alliance 6.7 system and NineAlliance 4.7 17.00 (uvitec).

### Statistical analysis

Statistical analyses were performed using Prism 7 for Mac OS X Version 7.0c (GraphPad Software Inc.). D’Agostino and Pearson normality test was carried out to determine normal distribution for each data. Differences in patient demographics, mRNA and protein expression between healthy and diseased cells or tissues, or by treatments were compared by unpaired t test when the data were normally distributed. Mann Whitney test was used when the data were not normally distributed or the numbers were small (less than 8). Statistical significance was set at p < 0.05.

## Supplementary information

Supplementary information

## Data Availability

The data supporting the findings of this study are available upon request from the corresponding author.

## References

[1] Disease GBD, Injury I, Prevalence C (2017). Global, regional, and national incidence, prevalence, and years lived with disability for 328 diseases and injuries for 195 countries, 1990–2016: a systematic analysis for the Global Burden of Disease Study 2016. Lancet.

[2] Global Burden of Disease Study, C. Global, regional, and national incidence, prevalence, and years lived with disability for 301 acute and chronic diseases and injuries in 188 countries, 1990–2013 (2015). a systematic analysis for the Global Burden of Disease Study 2013. Lancet.

[3] Dean BJF, Dakin SG, Millar NL, Carr AJ (2017). Review: Emerging concepts in the pathogenesis of tendinopathy. Surgeon.

[4] Kaux JF, Forthomme B, Goff CL, Crielaard JM, Croisier JL (2011). Current opinions on tendinopathy. J Sports Sci Med.

[5] McCormick, A. *et al*. Morbidity statistics from general practice: fourth national study, 1991–92. (HMSO, 1995).

[6] Gaida JE, Alfredson H, Kiss ZS, Bass SL, Cook JL (2010). Asymptomatic Achilles tendon pathology is associated with a central fat distribution in men and a peripheral fat distribution in women: a cross sectional study of 298 individuals. BMC Musculoskelet Disord.

[7] James SL, Bates BT, Osternig LR (1978). Injuries to runners. Am J Sports Med.

[8] Dean BJ, Franklin SL, Carr AJ (2012). A systematic review of the histological and molecular changes in rotator cuff disease. Bone Joint Res.

[9] Neer CS (1972). Anterior acromioplasty for the chronic impingement syndrome in the shoulder: a preliminary report. J Bone Joint Surg Am.

[10] Gerber C, Schneeberger AG, Hoppeler H, Meyer DC (2007). Correlation of atrophy and fatty infiltration on strength and integrity of rotator cuff repairs: a study in thirteen patients. J Shoulder Elbow Surg.

[11] Biernacka A, Dobaczewski M, Frangogiannis NG (2011). TGF-beta signaling in fibrosis. Growth Factors.

[12] Blobe GC, Schiemann WP, Lodish HF (2000). Role of transforming growth factor beta in human disease. N Engl J Med.

[13] Morita W, Snelling SJ, Dakin SG, Carr AJ (2016). Profibrotic mediators in tendon disease: a systematic review. Arthritis Res Ther.

[14] Wynn TA (2008). Cellular and molecular mechanisms of fibrosis. J Pathol.

[15] Millar NL, Dean BJ, Dakin SG (2016). Inflammation and the continuum model: time to acknowledge the molecular era of tendinopathy. Br J Sports Med.

[16] Dakin SG (2018). Pathogenic stromal cells as therapeutic targets in joint inflammation. Nat Rev Rheumatol.

[17] Morita W, Dakin SG, Snelling SJB, Carr AJ (2017). Cytokines in tendon disease: A Systematic Review. Bone Joint Res.

[18] Tohyama H, Yasuda K, Uchida H, Nishihira J (2007). The responses of extrinsic fibroblasts infiltrating the devitalised patellar tendon to IL-1beta are different from those of normal tendon fibroblasts. J Bone Joint Surg Br.

[19] Dakin SG (2015). Inflammation activation and resolution in human tendon disease. Sci Transl Med.

[20] Dakin SG (2017). Persistent stromal fibroblast activation is present in chronic tendinopathy. Arthritis Res Ther.

[21] Bergqvist F (2019). Divergent roles of prostacyclin and PGE2 in human tendinopathy. Arthritis Res Ther.

[22] Weber A, Wasiliew P, Kracht M (2010). Interleukin-1 (IL-1) pathway. Sci Signal.

[23] Scott A (2007). Tenocyte responses to mechanical loading *in vivo*: a role for local insulin-like growth factor 1 signaling in early tendinosis in rats. Arthritis Rheum.

[24] Chen MH, Huang YC, Sun JS, Chao YH, Chen MH (2015). Second messengers mediating the proliferation and collagen synthesis of tenocytes induced by low-level laser irradiation. Lasers Med Sci.

[25] Dudley DT, Pang L, Decker SJ, Bridges AJ, Saltiel AR (1995). A synthetic inhibitor of the mitogen-activated protein kinase cascade. Proc Natl Acad Sci USA.

[26] Feng JQ (2003). NF-kappaB specifically activates BMP-2 gene expression in growth plate chondrocytes *in vivo* and in a chondrocyte cell line *in vitro*. J Biol Chem.

[27] Feng X (2013). TNF-alpha triggers osteogenic differentiation of human dental pulp stem cells via the NF-kappaB signalling pathway. Cell Biol Int.

[28] Fukui N (2006). Pro-inflammatory cytokine tumor necrosis factor-alpha induces bone morphogenetic protein-2 in chondrocytes via mRNA stabilization and transcriptional up-regulation. J Biol Chem.

[29] Ibarra Urizar A, Friberg J, Christensen DP, Lund Christensen G, Billestrup N (2016). Inflammatory Cytokines Stimulate Bone Morphogenetic Protein-2 Expression and Release from Pancreatic Beta Cells. J Interferon Cytokine Res.

[30] Dennler S (1998). Direct binding of Smad3 and Smad4 to critical TGF beta-inducible elements in the promoter of human plasminogen activator inhibitor-type 1 gene. EMBO J.

[31] Korchynskyi O, ten Dijke P (2002). Identification and functional characterization of distinct critically important bone morphogenetic protein-specific response elements in the Id1 promoter. J Biol Chem.

[32] Ghosh AK, Vaughan DE (2012). PAI-1 in tissue fibrosis. J Cell Physiol.

[33] Edhayan G (2016). Inflammatory properties of inhibitor of DNA binding 1 secreted by synovial fibroblasts in rheumatoid arthritis. Arthritis Res Ther.

[34] Je YJ (2014). Inhibitory role of Id1 on TGF-beta-induced collagen expression in human dermal fibroblasts. Biochem Biophys Res Commun.

[35] Carter AB, Hunninghake GW (2000). A constitutive active MEK–> ERK pathway negatively regulates NF-kappa B-dependent gene expression by modulating TATA-binding protein phosphorylation. J Biol Chem.

[36] Goodier HC (2016). Comparison of transforming growth factor beta expression in healthy and diseased human tendon. Arthritis Res Ther.

[37] Bitzer M (2000). A mechanism of suppression of TGF-beta/SMAD signaling by NF-kappa B/RelA. Genes Dev.

[38] Yamazaki M (2009). Tumor necrosis factor alpha represses bone morphogenetic protein (BMP) signaling by interfering with the DNA binding of Smads through the activation of NF-kappaB. J Biol Chem.

[39] Cushing MC, Mariner PD, Liao JT, Sims EA, Anseth KS (2008). Fibroblast growth factor represses Smad-mediated myofibroblast activation in aortic valvular interstitial cells. FASEB J.

[40] Lee JS, Ha L, Park JH, Lim JY (2012). Mechanical stretch suppresses BMP4 induction of stem cell adipogenesis via upregulating ERK but not through downregulating Smad or p38. Biochem Biophys Res Commun.

[41] Sapkota G (2007). signaling through integrated inputs into the Smad1 linker. Mol Cell.

[42] Mukai T (2007). TNF-alpha inhibits BMP-induced osteoblast differentiation through activating SAPK/JNK signaling. Biochem Biophys Res Commun.

[43] Kretzschmar M, Doody J, Timokhina I, Massague J (1999). A mechanism of repression of TGFbeta/ Smad signaling by oncogenic Ras. Genes Dev.

[44] Liu X, Hubchak SC, Browne JA, Schnaper HW (2014). Epidermal growth factor inhibits transforming growth factor-beta-induced fibrogenic differentiation marker expression through ERK activation. Cell Signal.

[45] Lo RS, Wotton D, Massague J (2001). Epidermal growth factor signaling via Ras controls the Smad transcriptional co-repressor TGIF. EMBO J.

[46] Attisano L, Wrana JL (2002). Signal transduction by the TGF-beta superfamily. Science.

[47] Derynck R, Zhang YE (2003). Smad-dependent and Smad-independent pathways in TGF-beta family signalling. Nature.

[48] Hayashida T, Decaestecker M, Schnaper HW (2003). Cross-talk between ERK MAP kinase and Smad signaling pathways enhances TGF-beta-dependent responses in human mesangial cells. FASEB J.

[49] Wurgler-Hauri CC, Dourte LM, Baradet TC, Williams GR, Soslowsky LJ (2007). Temporal expression of 8 growth factors in tendon-to-bone healing in a rat supraspinatus model. J Shoulder Elbow Surg.

[50] Leask A, Parapuram SK, Shi-Wen X, Abraham DJ (2009). Connective tissue growth factor (CTGF, CCN2) gene regulation: a potent clinical bio-marker of fibroproliferative disease?. J Cell Commun Signal.

[51] Phanish MK, Winn SK, Dockrell ME (2010). Connective tissue growth factor-(CTGF, CCN2)–a marker, mediator and therapeutic target for renal fibrosis. Nephron Exp Nephrol.

[52] George J, Tsutsumi M (2007). siRNA-mediated knockdown of connective tissue growth factor prevents N-nitrosodimethylamine-induced hepatic fibrosis in rats. Gene Ther.

[53] Fedorczyk JM (2010). Exposure-dependent increases in IL-1beta, substance P, CTGF, and tendinosis in flexor digitorum tendons with upper extremity repetitive strain injury. J Orthop Res.

[54] Lu HK, Tseng CC, Lee YH, Li CL, Wang LF (2010). Flutamide inhibits nifedipine- and interleukin-1 beta-induced collagen overproduction in gingival fibroblasts. J Periodontal Res.

[55] Nowinski D (2010). Inhibition of connective tissue growth factor/CCN2 expression in human dermal fibroblasts by interleukin-1alpha and beta. J Cell Biochem.

[56] Sanchez-Lopez E (2008). Inhibitory effect of interleukin-1beta on angiotensin II-induced connective tissue growth factor and type IV collagen production in cultured mesangial cells. Am J Physiol Renal Physiol.

[57] Lui PP, Chan KM (2011). Tendon-derived stem cells (TDSCs): from basic science to potential roles in tendon pathology and tissue engineering applications. Stem Cell Rev Rep.

[58] Bi Y (2007). Identification of tendon stem/progenitor cells and the role of the extracellular matrix in their niche. Nat Med.

[59] Rui YF (2010). Isolation and characterization of multipotent rat tendon-derived stem cells. Tissue Eng Part A.

[60] Cagnol S, Chambard JC (2010). ERK and cell death: mechanisms of ERK-induced cell death–apoptosis, autophagy and senescence. FEBS J.

[61] Kauppinen A, Suuronen T, Ojala J, Kaarniranta K, Salminen A (2013). Antagonistic crosstalk between NF-kappaB and SIRT1 in the regulation of inflammation and metabolic disorders. Cell Signal.

[62] Franceschi C, Campisi J (2014). Chronic inflammation (inflammaging) and its potential contribution to age-associated diseases. J Gerontol A Biol Sci Med Sci.

[63] Dinarello CA (2018). Overview of the IL-1 family in innate inflammation and acquired immunity. Immunol Rev.

[64] Millar NL (2010). Inflammation is present in early human tendinopathy. Am J Sports Med.

[65] Klatte-Schulz Franka, Minkwitz Susann, Schmock Aysha, Bormann Nicole, Kurtoglu Alper, Tsitsilonis Serafeim, Manegold Sebastian, Wildemann Britt (2018). Different Achilles Tendon Pathologies Show Distinct Histological and Molecular Characteristics. International Journal of Molecular Sciences.

[66] Murphy RJ, Daines MT, Carr AJ, Rees JL (2013). An Independent Learning Method for Orthopaedic Surgeons Performing Shoulder Ultrasound to Identify Full-Thickness Tears of the Rotator Cuff. Journal of Bone and Joint Surgery-American Volume.

[67] Post, M., Silver, R. & Singh, M. Rotator cuff tear. *Diagnosis and treatment. Clin Orthop Relat Res*, 78–91 (1983). 6825349

[68] Murphy RJ (2013). A Novel Minimally Invasive Ultrasound-Guided Technique to Biopsy Supraspinatus Tendon. Oper Tech Orthop.

[69] Franklin SL (2014). Up-regulation of Glutamate in Painful Human Supraspinatus Tendon Tears. Am J Sports Med.

[70] Rizzardi AE (2012). Quantitative comparison of immunohistochemical staining measured by digital image analysis versus pathologist visual scoring. Diagn Pathol.

[71] Ruifrok AC, Johnston DA (2001). Quantification of histochemical staining by color deconvolution. Anal Quant Cytol Histol.

[72] Poulsen RC, Watts AC, Murphy RJ, Carr AJ, Hulley PA (2013). Ageing and Osteoarthritis Markers Identified by Maldi Imaging Mass Spectrometry. Osteoarthr Cartilage.

[73] Dakin SG (2018). Chronic inflammation is a feature of Achilles tendinopathy and rupture. Br J Sports Med.

[74] Gingery A (2014). TGF-beta signaling regulates fibrotic expression and activity in carpal tunnel syndrome. J Orthop Res.

[75] Schmittgen TD, Livak KJ (2008). Analyzing real-time PCR data by the comparative C(T) method. Nat Protoc.

[76] Vandesompele J (2002). Accurate normalization of real-time quantitative RT-PCR data by geometric averaging of multiple internal control genes. Genome Biol.

