# Supplementary material for: ERK1/2 drives IL-1β-induced expression of TGF-β1 and BMP-2 in torn tendons
Source: Sci Rep. 2019 Dec 12;9:19005. doi: 10.1038/s41598-019-55387-1 (PMC6908634; doi:10.1038/s41598-019-55387-1)
Supplement: Supplementary file 1 — Supplementary information [file 41598_2019_55387_MOESM1_ESM.pdf]

Supplementary Information

Title: ERK1/2 drives IL-1β-induced expression of TGF-β1 and BMP-2 in torn tendons

Authors: Wataru Morita*[1], Sarah JB Snelling[1], Kim Wheway[1], Bridget Watkins[1], Louise Appleton[1], Andrew J Carr[1+] and Stephanie G Dakin**[1+]

* denotes primary corresponding author

** denotes secondary corresponding author

[+] denotes equal senior author contribution

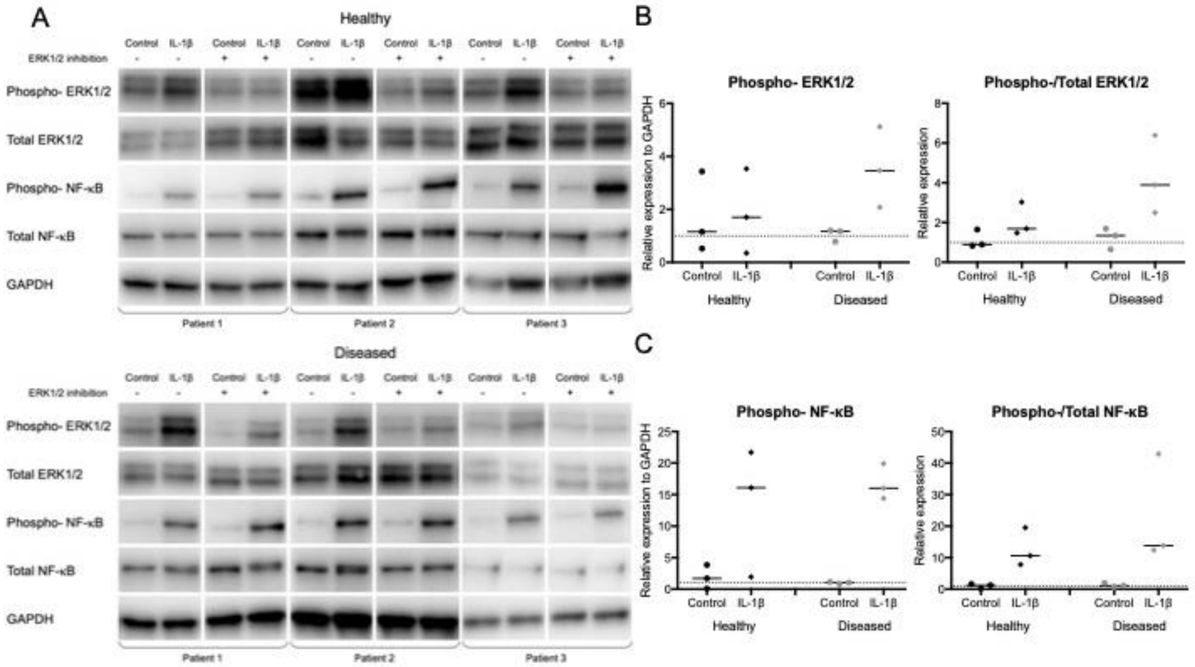

SUPPLEMENTARY FIGURE 1: Western blotting showing cell signalling activation of ERK1/2 and NF-κB in healthy and diseased tendon stromal cells after treatment by IL-1β or vehicle control (control) with or without ERK1/2 inhibition. (A) All obtained images of Western blotting for phospho- ERK1/2, total ERK1/2, phospho- NF-κB, total NF-κB and GAPDH in healthy (N = 3) and diseased (N = 3) cells are shown. Western blotting also confirms inhibition of the ERK1/2 signalling pathway by the small molecule inhibitor. Levels of pathway activations in healthy and diseased cells were quantified by measuring the intensities of Western blots (see Supplementary Method 2) and normalised to the expression of GAPDH or to the respective total protein for (B) ERK1/2 and (C) NF-κB. Bars shown represent median.

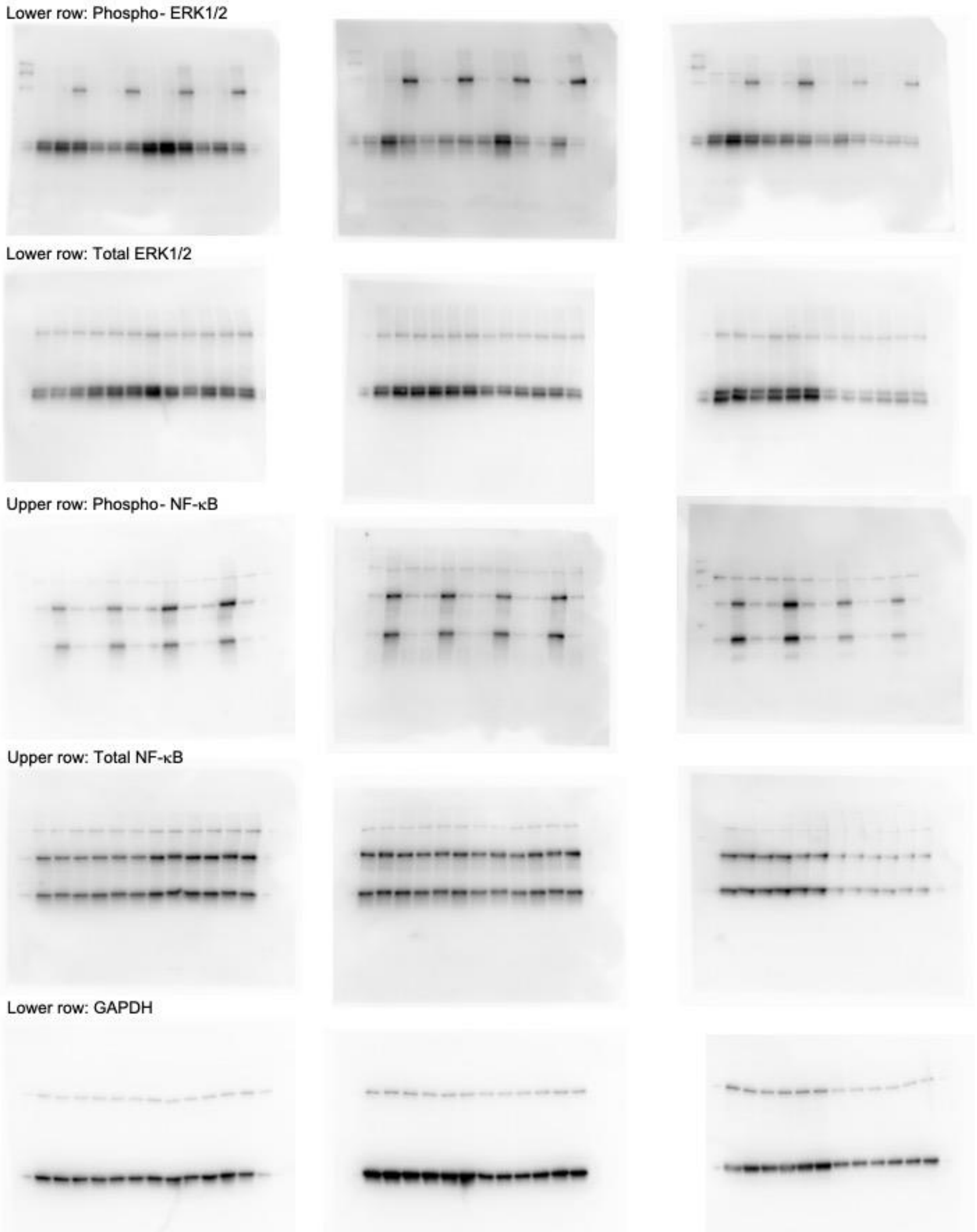

SUPPLEMENTARY FIGURE 1': Full-length Western blotting showing cell signalling activation of ERK1/2 and NF-κB in healthy and diseased tendon stromal cells after treatment by IL-1β or vehicle control (control) with or without ERK1/2 inhibition. Only the blots for the indicated rows and lanes #1, #2, #4 and #5 were used for this study. For each donor, cells were treated with vehicle control for lanes #1 and #4 and IL-1β for lanes #2 and #5. The ERK1/2 inhibitor was added to lanes #4 to #5.

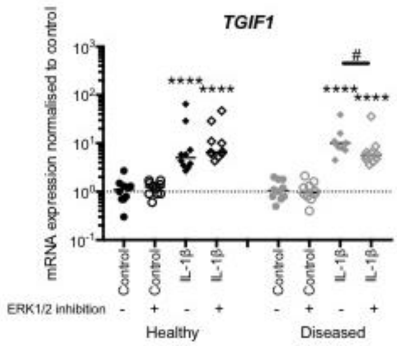

SUPPLEMENTARY FIGURE 2: mRNA expression of *TGIF1* in stromal cells derived from healthy and diseased tendons after treatment by IL-1β or vehicle control (control) with or without ERK1/2 inhibition. Levels of mRNA expression in healthy (n=10) and diseased (n=10) cells were quantified by RT-qPCR. IL-1β treatment induced *TGIF1* mRNA expression in both cells. ERK1/2 inhibition partially suppressed IL-1β-induced *TGIF1* mRNA expression in the diseased cells. Bars shown represent median. * indicates significant difference to the respective vehicle control. # indicates significant difference between with and without ERK1/2 inhibition. */# $P < 0.05$, ** $P < 0.01$, *** $P < 0.001$, **** $P < 0.0001$.

SUPPLEMENTARY TABLE 1: Details of primers used

Gene description	Gene symbol	Manufacturer	Reference or forward/reverse primer sequence
Transforming growth factor beta 1	*TGFB1*	QIAGEN	HS_TGFB1_1_SG (QT00000728)
Transforming growth factor beta receptor 1	*TGFBR1*	QIAGEN	HS_TGFBR1_1_SG (QT00083412)
Transforming growth factor beta receptor 2	*TGFBR2*	QIAGEN	HS_TGFBR2_1_SG (QT00014350)
Connective tissue growth factor	*CTGF*	QIAGEN	HS_CTGF_1_SG (QT00052899)
Bone morphogenetic protein 2	*BMP2*	QIAGEN	HS_BMP2_1_SG (QT00012544)
Bone morphogenetic protein 7	*BMP7*	QIAGEN	HS_ BMP7_1_SG (QT00068936)
Growth differentiation factor 7	*GDF7 (BMP12)*	QIAGEN	HS_ BMP12_1_SG (QT00227507)
Serpine family E member 1	*SERPINE1*	QIAGEN	HS_SERPINE1_1_SG (QT00062496)
Inhibitor of DNA binding 1	*ID1*	QIAGEN	HS_ID1_1_SG (QT00230650)
Prostaglandin-endoperoxide synthase 2	*PGS2 (COX2)*	Primerdesign	CAGGCTTCCATTGACCAGAG / TTTCTCCTGTAAGTTCTTCAAATGAT
Prostaglandin E synthase	*PTGES*	QIAGEN	HS_PTGES_1_SG (QT00208607)
Actin, beta	*ACTB*	QIAGEN	HS_ACTB_1_SG (QT00095431)
Glyceraldehyde-3-phosphate dehydrogenase	*GAPDH*	QIAGEN	HS_GAPDH_1_SG (QT00079247)

SUPPLEMENTARY TABLE 2: Details of antibodies used for Western blotting

Antibody	Species	Isotype	Dilution	Manufacturer	Reference
Phospho- Erk1/2	rabbit	IgG	1:1000	Cell Signaling	4370
Total Erk1/2	rabbit	IgG	1:1000	Cell Signaling	4695
Phospho- NF-κB (p65)	rabbit	IgG	1:1000	Cell Signaling	3033
Total NF-κB (p65)	rabbit	IgG	1:1000	Cell Signaling	8242
GAPDH	rabbit	IgG	1:1000	Cell Signaling	5174
Anti-rabbit IgG	Not applicable	polyclonal	1:1000	Cell Signaling	7074

Supplementary Method 1: Immunohistochemistry Image Analysis Macros

- Nuclei Count

```
imgName=getTitle();run("Colour Deconvolution", "vectors=[H&E 2]");
selectWindow(imgName+"-(Colour_1)"); setAutoThreshold("Default");
//run("Threshold..."); setThreshold(0, *); run("Convert to Mask"); run("Fill Holes");
run("Despeckle"); run("Remove Outliers...", "radius=10 threshold=50 which=Dark");
run("Analyze Particles...", "size=10-Infinity circularity=0.00-1.00 show=Nothing display
summarize");
```

* manually set threshold for each dataset

- Immunopositive Cell Count

```
imgName=getTitle(); run("Colour Deconvolution", "vectors=[H DAB]");
selectWindow(imgName+"-(Colour_2)"); setAutoThreshold("Default");
//run("Threshold..."); setThreshold(0, *); run("Convert to Mask"); run("Remove
Outliers...", "radius=10  threshold=50 which=Dark"); run("Analyze Particles...",
"size=10-Infinity circularity=0.00-1.00 show=Nothing display summarize");
```

* manually set threshold for each dataset

Supplementary Method 2: Semi-quantitative Analysis of Western Blotting

Intensity of the protein bands acquired by Western blotting were quantified by ImageJ (1.47v, National Institutes of Health); the Gel Analyzer command was used to generate profile plots of the intensity of the bands. The areas of each region of interest for each band on the blots were measured as values that represent the intensities. Data were normalised to the median of the control group and the relative values of the phosphorylated proteins of interest to their respective total proteins or housekeepers were calculated.

Supplementary Method 3: Validation Experiment for the IL-1β Treatment Dosage

Following serum-starving overnight, healthy cells cultured up to passage 3 (N = 3) were seeded in 24-well plates, grown to sub-confluence and treated with human recombinant IL-1β (0.1, 1, 5 or 50 ng/ml) or vehicle control for 24 hours. IL-1β treatment did not significantly modulate *TGFB1* and *BMP2* mRNA expression compared to vehicle control regardless of the dose. However, IL-1β treatment tended to induce *BMP2* mRNA expression compared to vehicle control at all dosages.IL-1β treatment significantly suppressed *CTGF* mRNA expression compared to vehicle control at 5 ng/ml.

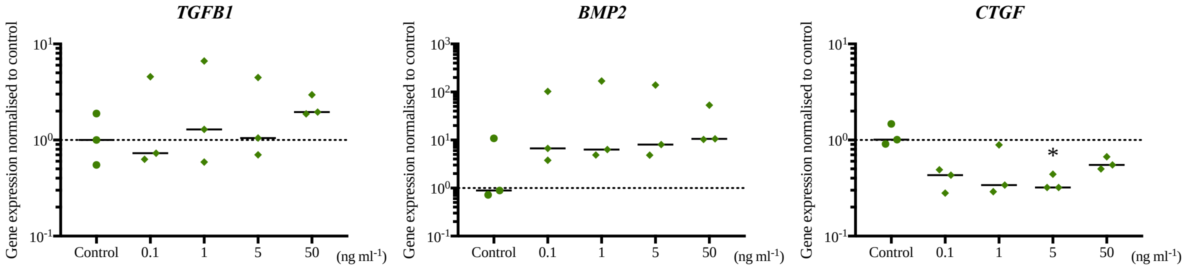

SUPPLEMENTARY FIGURE 3: mRNA expression of *TGFB1*, *BMP2* and *CTGF* in stromal cells derived from healthy tendons after IL-1β treatment or vehicle control (control). Healthy (N = 3) tendon stromal cells were treated with different doses of IL-1β or vehicle control and levels of mRNA expression were quantified by RT-qPCR. Bars shown represent median. * indicates significant difference to vehicle control. *=p<0.05.
